# Carmustine-releasing wafers for brain metastasis: single institution experience and results of the VIGILANT registry

**DOI:** 10.1007/s11060-026-05871-2

**Published:** 2026-11-11

**Authors:** Eleanor C. Smith, Jared M. Kaplan, Bryan T. Mott, Carol Kittel, Molly Ehrig, Christina K. Cramer, Jaclyn J. White, Stephen B. Tatter

**Affiliations:** 1 Department of Neurological Surgery, Wake Forest University School of Medicine, 1 Medical Center Blvd, Winston-Salem, NC 27157 USA; 2 Division of Public Health Sciences, Department of Biostatistics and Data Science, Wake Forest University School of Medicine, Winston-Salem, NC USA; 3 Department of Radiation Oncology, Wake Forest University School of Medicine, Winston- Salem, NC USA

**Keywords:** Carmustine, Brain metastasis, Radiosurgery, Tumor recurrence

## Abstract

**Purpose:**

Brain metastases affect many patients with solid tumor malignancies. Treatments include surgery, radiation, immunotherapy, and CNS-penetrating chemotherapy. Nonetheless, rates of CNS-related tumor death remain high. Local recurrence after radiosurgery and lesions with a V12 too high for radiosurgery present a particular challenge. The VIGILANT (Vigilant Observation of GLIADEL Wafer Implant) registry prospectively examined use and efficacy of carmustine-releasing wafers which includes patients with brain metastases [1]. Here we present a retrospective analysis of patients with brain metastasis from the VIGILANT registry.

**Methods:**

Forty-seven patients were observed after surgical resection of brain metastasis and GLIADEL implantation at Wake Forest Baptist Medical Center. Thirty-eight patients underwent treatment for local recurrence following radiosurgery. Nine had lesions treated that were deemed too large for successful primary radiosurgery. Patients were evaluated primarily for overall survival (OS) and progression free survival (PFS) from the date of surgery, and secondarily for adverse events and local progression.

**Results:**

Median OS was 23 months. Of 25 deaths, 7 (28%) met criteria for CNS-tumor related etiology. 24 (51.1%) patients had progression during follow up, 6 (12.8%) had local progression. 2 (4.3%) overall had local progression that required additional neurosurgical treatment. Of 9 patients not previously treated with radiosurgery, 4 (44.4%) had no progression. Of 5 patients who received GLIADEL as their upfront cranial intervention, none had local recurrence. 43 patients had adverse events; 2 considered possibly GLIADEL related.

**Conclusions:**

In this single-institution retrospective analysis of prospectively collected registry data, patients undergoing resection and carmustine wafer implantation after prior radiosurgery or for lesions considered unsuitable for primary radiosurgery had favorable observed survival (OS 23 months), intracranial progression (1-year local progression rate of 12%), and minimal related adverse-event outcomes.

**Supplementary Information:**

The online version contains supplementary material available at https://doi.org/10.1007/s11060-026-05871-2.

## Introduction

Brain metastases are the most common Central Nervous System (CNS) malignancy in adults, affecting approximately 30–40% of cancer patients with primary solid tumor malignancies [2, 3]. Lung cancer accounts for the majority of brain metastases, followed by breast cancer and melanoma [4, 5]. Rates of brain metastasis requiring treatment are increasing because of improved control of extracranial disease with systemic treatments leading to improved overall survival as well as increasing detection by improving imaging [3]. Currently, standard of care treatments for brain metastases include surgical resection, radiotherapy (both whole brain and stereotactic), as well as cytotoxic chemotherapies and targeted therapies. The latter often have varying degrees of efficacy due to limited ability to cross the blood brain barrier (BBB) or particularly resistant genetic alterations [2, 3, 6]. Despite this array of therapies, overall survival in patients diagnosed with brain metastasis remains poor. A meta-analysis of 5325 patients by Li et al. showed an overall survival of 17.9 months in patients with stable extracranial disease and 8.0 months for patients with progressive disease [7].

Patients with local recurrence after radiosurgery or with very large tumors are particularly problematic because of high rates of radiation toxicity from radiation retreatment or from treating large intracranial volumes.

To overcome the BBB, alternative routes of administration of chemotherapeutics have been employed, including direct implantation into brain tumor surgical cavities. GLIADEL→ is a biodegradable carmustine chemotherapy wafer shown to deliver high doses of carmustine to a resection cavity, with limited distant penetration to normal brain tissues [8]. Approved by the US Food and Drug Administration for use in 1996, GLIADEL, an implantable carmustine wafer, has shown efficacy in treatment of recurrent malignant glioma with an acceptable side-effect profile [8–10].

Retrospective series of GLIADEL for treatment of brain metastases from solid tumors show there may be decreased local recurrence [9–14]. The VIGILANT registry (Vigilant Observation of Gliadel Wafer Implant, NCT02684838, Registered February 2, 2016), initiated in 2017, aimed to prospectively examine the use and efficacy of GLIADEL wafers in the USA and includes a subset of patients with brain metastases enrolled at Wake Forest Baptist Medical Center [15]. Here we present a retrospective analysis of prospectively collected, single-institution cohort results from the VIGILANT registry of GLIADEL for treatment of patients with brain metastasis from solid tumors.

## Methods

Patients undergoing surgical resection of metastatic brain tumors were enrolled at Wake Forest Baptist Medical Center from 2017 to 2023 with a follow up period of 3 years. The protocol was reviewed and approved by the WFU (Wake Forest University) Health Sciences Institutional Review Board and performed in accordance with the ethical standards outlined in the 1964 Declaration of Helsinki. Informed consent to participate was obtained from each patient or the patient’s legally appointed representative. Management of the registry was overseen by Azurity Pharmaceuticals, previously Arbor Pharmaceuticals. No sites other than Wake Forest Baptist Medical Center enrolled patients with brain metastases in the VIGILANT registry (NCT02684838, registered February, 2016) [1]; therefore, this report represents a retrospective analysis of results from a single institution.

Inclusion criteria included patients > 18 years of age, eligible to receive GLIADEL for treatment of a CNS malignancy, and no medical comorbidities that increased patient risk in the registry. Patients included in this analysis were limited to those undergoing GLIADEL wafer implantation as part of their care for treatment of CNS metastasis from solid tumor primary malignancy. Patients were determined unsuitable for primary SRS based on the V12 or had limitations preventing them from receiving radiation as determined by the treating radiation oncologist.

As part of the VIGILANT registry, patients were followed up in 3-month (+/- 1 month) intervals with diagnostic imaging obtained (MRI or CT scan) to assess for disease progression. Local recurrence in the study was defined as within approximately 2 cm of primary intracranial lesion and/or resection cavity, whereas distant recurrence was defined as > 2 cm intracranially.

Primary objectives include evaluating the overall survival (OS) and progression free survival (PFS) of patients treated with GLIADEL wafers. Secondary objectives include evaluating adverse events related to GLIADEL wafer use in patients with metastasis from solid tumor primary malignancy.

### Statistical analysis

Descriptive statistics were summarized by frequency (percent) or median [IQR]. Two-tailed p-values of less than 0.05 were considered statistically significant for this study. To evaluate associations between overall survival and age, sex, race, dose, residual tumor volume, baseline tumor volume, prior treatment, degree of resection, and pathology (lung, breast, or other), univariate Cox proportional hazards regression models were used. The proportional hazards assumption was assessed and held for each predictor. Multivariate Cox proportional hazard regression modeling was performed using backward covariate selection determined by Akaike information criterion (AIC) selection criteria with final model selection made by log likelihood statistics. Estimates for freedom from overall survival were obtained with the Kaplan-Meier method with censoring performed at study end. Median survival time for patients whose death was considered CNS-tumor related was calculated by summarizing time to event for this subset of patients only.

To investigate associations between age, sex, race, dose, residual volume, tumor volume, prior treatment, degree of resection pathology (lung, breast, or other) and local progression, univariate competing risks regression models were used with death considered the competing event. Multivariate competing risk regression modeling was performed with no covariates remaining after backward selection as determined by Akaike information criterion (AIC) selection criteria. The Fine and Gray method was used for estimating cumulative incidence of progression while accounting for death as a competing risk.

Among the subset of patients who underwent prior stereotactic radiosurgery (SRS), the Fine and Gray method was used for estimating cumulative incidence of local progression while accounting for death as a competing risk.

## Results

**Fig. 1:**
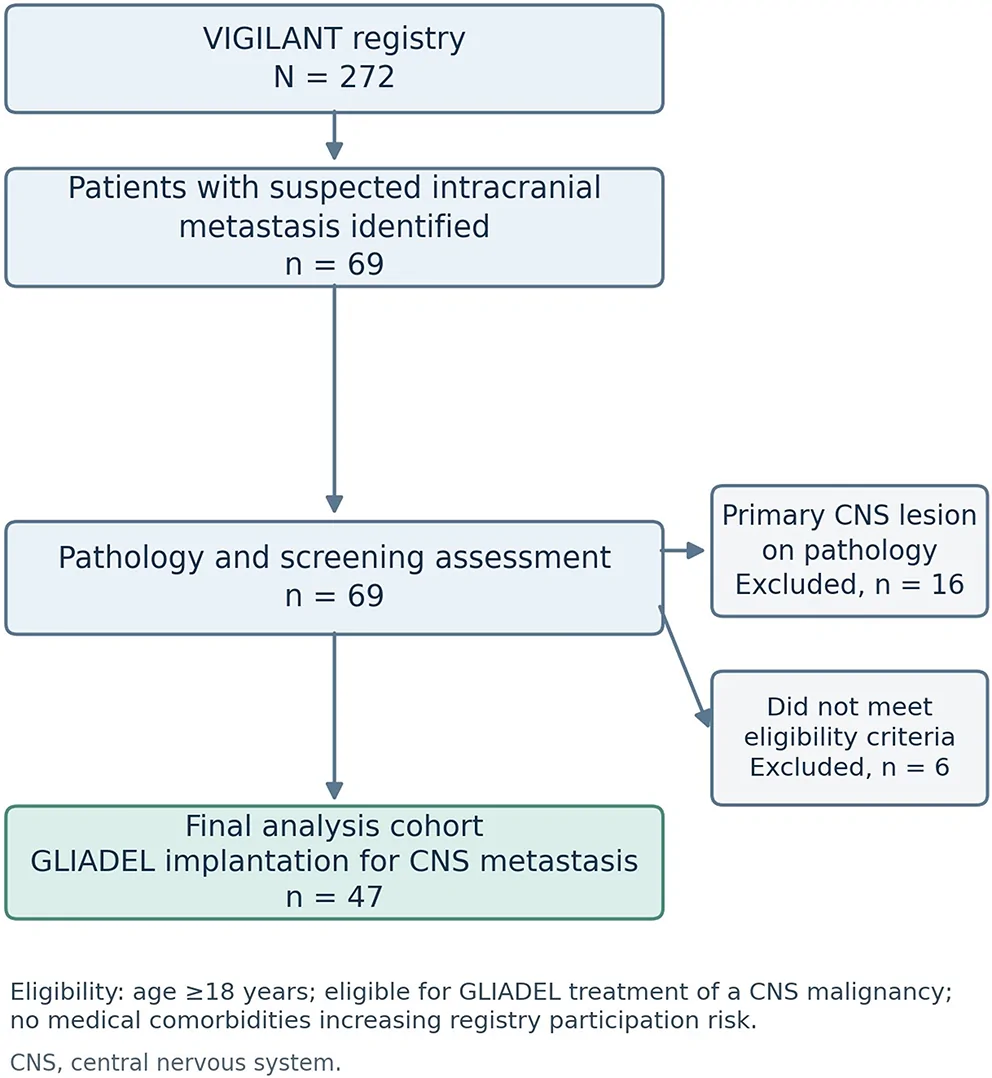
Figure 1

Of the 272 patients within the VIGILANT registry, a total of 69 patients with multiple intracranial lesions were identified and consented. Amongst that cohort, 16 patients were excluded from analysis due to pathology showing primary CNS lesion, and 6 patients were deemed to not be eligible candidates for GLIADEL wafer placement due to screening failure. Therefore, 47 patients were included in the final analysis as demonstrated in Fig. 1.

Patient descriptives are presented in Table 1. 29 patients were female, and 18 were male. Median age was 64 (IQR 56.1, 68.4) at diagnosis. 25 patients had pathology consistent with lung cancer including 10 lung adenocarcinoma, 4 small cell lung cancer (SCLC), 1 neuroendocrine, 1 carcinoid, and 9 otherwise unspecified non-small cell lung cancer (NSCLC). Other pathologies included breast (8 patients), colorectal (3 patients), esophageal (2 patients), melanoma (2 patients), squamous cell carcinoma (2 patients), renal cell carcinoma (1 patient), bladder (1 patient), endometrial (1 patient), mediastinal adenocarcinoma (1 patient), adenoid cystic carcinoma (1 patient). 38 patients were treated for local recurrence despite SRS while 9 patients had lesions with a V12 deemed too high for successful primary SRS.

**Table 1:** Patient descriptives

	Overall (*N* = 47)
**Age at surgery**	
Median [Q1, Q3]	64.0 [56.1, 68.4]
**Sex**	
Female	29 (61.7%)
Male	18 (38.3%)
**Race**	
Black	8 (17.0%)
White	39 (83.0%)
**Dose (# of wafers)**	
Median [Q1, Q3]	6.00 [4.50, 8.00]
**Residual volume (mm** ^**3**^ **)**	
Median [Q1, Q3]	0 [0, 0]
Missing	1 (2.1%)
**Tumor volume (mm** ^**3**^ **)**	
Median [Q1, Q3]	7360 [3620, 13700]
**Prior treatment (intracranial)**	
N	5 (10.6%)
Y	42 (89.4%)
**SRS**	
N	9 (19.1%)
Y	38 (80.9%)
**Degree of resection**	
Gross Total	38 (80.9%)
Subtotal	8 (17.0%)
Missing	1 (2.1%)
**Progression**	
N	23 (48.9%)
Y	24 (51.1%)
**Pathology**	
Breast	8 (17.0%)
Lung	25 (53.2%)
Other	14 (29.8%)
**End of study reason**	
Investigator Decision	6 (12.8%)
Lost to Follow-Up	3 (6.4%)
Patient Death	25 (53.2%)
Study Completion	12 (25.5%)
Withdrawal of Consent	1 (2.1%)

Median tumor volume was 7360 mm^3^ (IQR 3620, 13700), with residual volume following resection ranging from 0 to 3.5 mm^3^ as assessed by postbaseline MRI. The median number of GLIADEL wafers placed was 6 (IQR 4.50, 8.00). Of the 47 patients in the study, 25 died and 22 were censored. 7 of these deaths met criteria for CNS-tumor related death, 13 due to extra-cranial disease progression, and for 5 patients the cause of death was unknown. 24 patients had progression, 13 did not have progression but died, and 10 did not have progression and did not die. 43 total patients had adverse events; two considered possibly GLIADEL related (which can be found detailed in Supplementary Table 1).

**Fig. 2:**
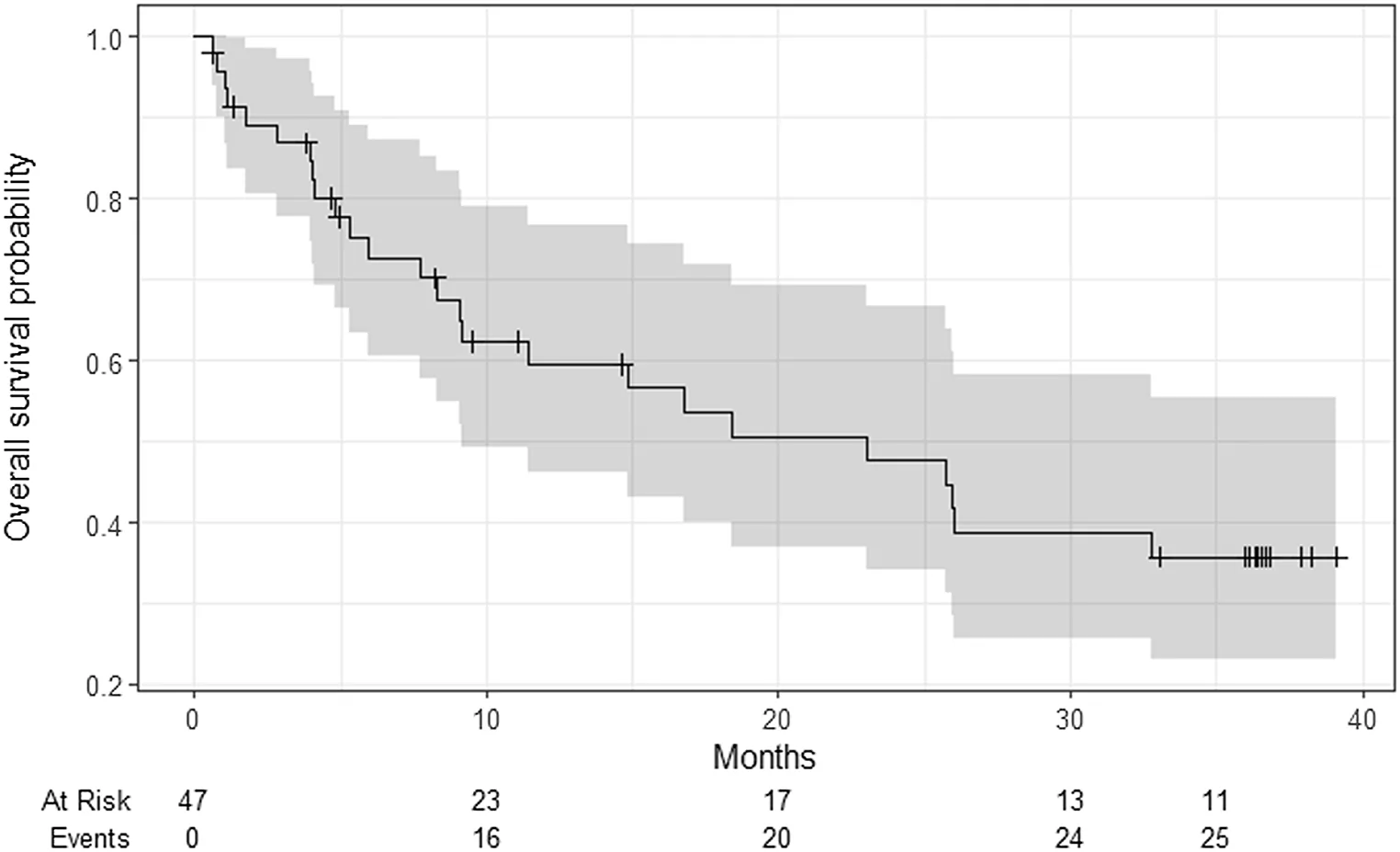
Survival plot

Median overall survival was 23 months. Overall survival at 6 and 12 months were 73% and 60%, respectively. Of 7 patients with death considered CNS-tumor related, median OS was 5.3 months. Age, sex, dose, residual volume, tumor volume, prior treatment, degree of resection and pathology were not found to be significantly associated with overall survival. Overall survival probability is shown in Fig. 2.

**Table 2:** Split by overall survival status

	Censored (*N* = 22)	Death (*N* = 25)	Overall (*N* = 47)	
**Age at surgery**				
Median [Q1, Q3]	60.9 [51.3, 67.0]	67.1 [61.3, 69.0]	64.0 [56.1, 68.4]	
**Sex**				
Female	14 (63.6%)	15 (60.0%)	29 (61.7%)	
Male	8 (36.4%)	10 (40.0%)	18 (38.3%)	
**Race**				
Black	5 (22.7%)	3 (12.0%)	8 (17.0%)	
White	17 (77.3%)	22 (88.0%)	39 (83.0%)	
**Dose**				
Median [Q1, Q3]	5.00 [4.00, 8.00]	8.00 [5.00, 8.00]	6.00 [4.50, 8.00]	
**Residual volume (mm** ^**3**^ **)**				
Median [Q1, Q3]	0 [0, 0]	0 [0, 0]	0 [0, 0]	
Missing	1 (4.5%)	0 (0%)	1 (2.1%)	
**Tumor volume (mm** ^**3**^ **)**				
Median [Q1, Q3]	5640 [1790, 12400]	8020 [5260, 16400]	7360 [3620, 13700]	
**Prior treatment (intracranial)**				
No	4 (18.2%)	1 (4.0%)	5 (10.6%)	
Yes	18 (81.8%)	24 (96.0%)	42 (89.4%)	
**SRS**				
No	5 (22.7%)	4 (16.0%)	9 (19.1%)	
Yes	17 (77.3%)	21 (84.0%)	38 (80.9%)	
**Degree of resection**				
Gross Total	17 (77.3%)	21 (84.0%)	38 (80.9%)	
Subtotal	4 (18.2%)	4 (16.0%)	8 (17.0%)	
Missing	1 (4.5%)	0 (0%)	1 (2.1%)	
**Pathology**				
Breast	5 (22.7%)	3 (12.0%)	8 (17.0%)	
Lung	9 (40.9%)	16 (64.0%)	25 (53.2%)	
Other	8 (36.4%)	6 (24.0%)	14 (29.8%)	

For the 24 patients that had progression, the median time to progression is 3.4 months (IQR 2.1, 4.6). As death was treated as a competing event, the median progression free survival was 5.5 months. 6 (24%) had local progression, 3 (12%) of which had subtotal resection. 2 (8%) had local progression that required additional neurosurgical treatment. 15 (60%) patients had distant progression, and 3 (13%) had unknown location of progression. Patient descriptives split by overall survival status is presented in Table 2. Of 38 patients previously treated with SRS, 19 (51%) had progression and 5 (13%) had local progression. Of 9 patients not previously treated with radiosurgery, 5 (56%) had progression, none with local progression.

**Fig. 3:**
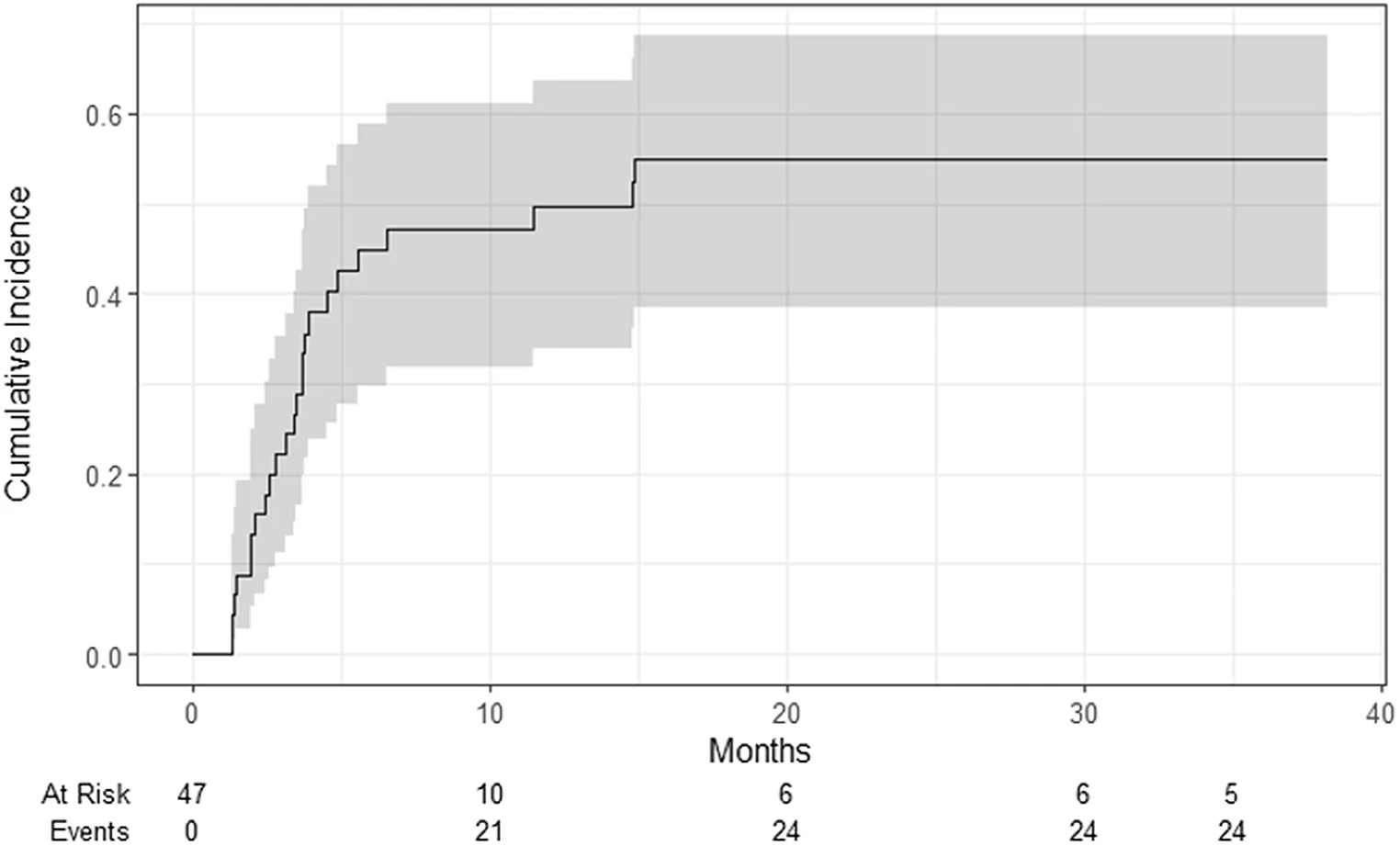
Cumulative incidence of progression estimates with 95% confidence intervals

Patient demographics and surgical characteristics are presented in Table 3. Just as with OS, no associations were found between the same variables and progression. Cumulative incidence of progression is shown in Fig. 3.

**Table 3:** Patient demographics and surgical characteristics by progression status

	Censored	Death	Progression	Overall
	(*N* = 10)	(*N* = 13)	(*N* = 24)	(*N* = 47)
**Age at surgery**				
Median [Q1, Q3]	57.7 [47.5, 65.8]	62.2 [59.9, 67.1]	67.1 [58.7, 68.9]	64.0 [56.1, 68.4]
**Sex**				
Female	7 (70.0%)	7 (53.8%)	15 (62.5%)	29 (61.7%)
Male	3 (30.0%)	6 (46.2%)	9 (37.5%)	18 (38.3%)
**Race**				
Black	3 (30.0%)	2 (15.4%)	3 (12.5%)	8 (17.0%)
White	7 (70.0%)	11 (84.6%)	21 (87.5%)	39 (83.0%)
**Dose**				
Median [Q1, Q3]	5.00 [4.00, 8.00]	8.00 [5.00, 8.00]	6.00 [5.00, 8.00]	6.00 [4.50, 8.00]
**Residual volume**				
Median [Q1, Q3]	0 [0, 0.560]	0 [0, 0]	0 [0, 0]	0 [0, 0]
Missing	1 (10.0%)	0 (0%)	0 (0%)	1 (2.1%)
**Tumor volume**				
Median [Q1, Q3]	8770 [4840, 13800]	9750 [3700, 16600]	6300 [3330, 10400]	7360 [3620, 13700]
**Prior treatment**				
N	2 (20.0%)	0 (0%)	3 (12.5%)	5 (10.6%)
Y	8 (80.0%)	13 (100%)	21 (87.5%)	42 (89.4%)
**SRS**				
N	3 (30.0%)	1 (7.7%)	5 (20.8%)	9 (19.1%)
Y	7 (70.0%)	12 (92.3%)	19 (79.2%)	38 (80.9%)
**Degree of resection**				
Gross Total	6 (60.0%)	12 (92.3%)	20 (83.3%)	38 (80.9%)
Subtotal	3 (30.0%)	1 (7.7%)	4 (16.7%)	8 (17.0%)
Missing	1 (10.0%)	0 (0%)	0 (0%)	1 (2.1%)
**Pathology**				
Breast	2 (20.0%)	1 (7.7%)	5 (20.8%)	8 (17.0%)
Lung	3 (30.0%)	8 (61.5%)	14 (58.3%)	25 (53.2%)
Other	5 (50.0%)	4 (30.8%)	5 (20.8%)	14 (29.8%)
**Adjuvant therapy**				
SRS	2 (20%)	2 (15.3%)	5 (21%)	9 (19.1%)
Chemo/Immunotherapy	1 (10%)	7 (53.8%)	3 (12.5%)	11 (23.4%)
SRS+Immunotherapy	1 (10%)	2 (15.3%)	0 (0%)	3 (6.4%)
No concurrent treatment	6 (60%)	2 (15.3%)	16 (66.6%)	24 (51%)

**Fig. 4:**
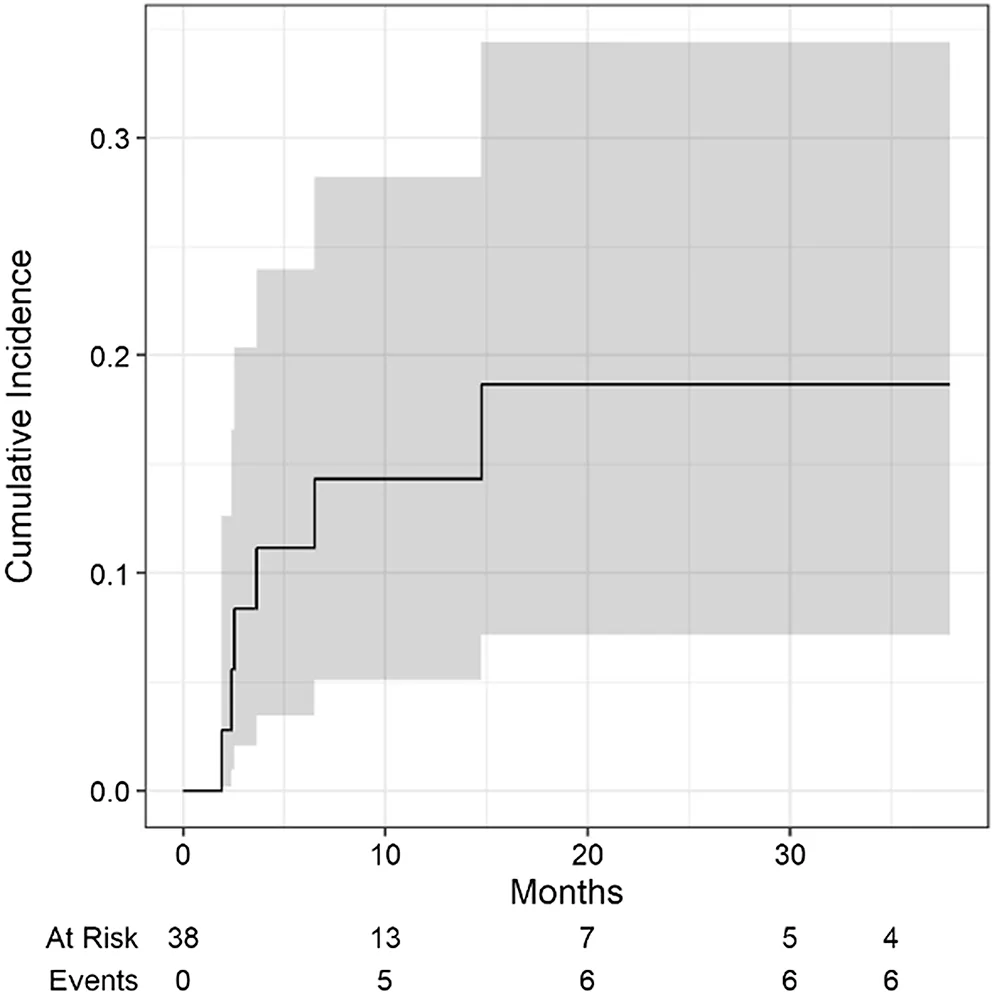
Cumulative incidence of local progression estimates with 95% confidence intervals

Cumulative incidence of local progression is shown in Fig. 4. Of the 5 patients who underwent SRS and had local progression, the median time to local progression was 3.1 months.

## Discussion

The present study focuses exclusively on a population with limited therapeutic alternatives. That includes patients whose brain metastases had recurred after stereotactic radiosurgery or whose presenting tumors were too large for resection followed by radiosurgery. This group faces high rates of local failure, and there is no consensus on optimal salvage therapy. Thus, the outcomes reported here provide valuable insight into a pressing clinical challenge and underscore the potential role of local carmustine delivery as a targeted intervention for improving disease control in patients without other attractive management options.

Treatment of brain metastases remains increasingly pertinent as growing numbers of patients with solid tumor malignancy are found to have brain metastases due to prolonged overall survival thanks to improving systemic therapies, and improving imaging modalities allowing earlier and more frequent diagnosis [4]. A series by Benna and colleagues estimated median survival of patients after primary diagnosis of brain metastases to be 4 months [16], with an estimated 23–46% of patients having recurrence after primary treatment within 7 months [17]. Of 5 patients in our cohort who had no prior intracranial treatment before GLIADEL placement, none had local progression which appears improved compared to estimated risk of progression in previously described cohorts. However, given the sample size would need further validation.

Carmustine wafers are employed for management of primary brain tumors, with FDA approval for treatment of recurrent high-grade gliomas in 1996, and initial treatment of high-grade gliomas in 2003 [18]. Randomized placebo-controlled trials have been performed evaluating efficacy of carmustine wafers in the treatment of primary and recurrent high-grade gliomas, both showing statistically significant survival benefit when compared to patients undergoing placebo wafer placement [19, 20]. Carmustine may still not penetrate deeply enough for most effective treatment of infiltrative glioma [21]. On the other hand, histological evaluation of brain metastases shows the maximum infiltration of all evaluated primary pathologies to be less than 2 mm into normal brain tissue, within the described volume of highest tissue dose of carmustine, which has been found to be approximately 3 mm from the implant in pharmacokinetic studies [8]. Despite these encouraging mechanistic considerations, a paucity of studies evaluate local drug delivery for brain metastasis.

A phase 2 clinical trial performed by Brem and colleagues published in 2013 evaluated the use of surgical resection and placement of carmustine wafers for upfront treatment of brain metastasis [22]. 59 patients were included in analysis, which predominantly aimed to evaluate neurocognitive function preservation in comparison to treatment with whole brain radiotherapy (WBRT). Secondary analysis evaluated disease control, which showed 28% of patients developed local recurrence and 48% of patients developed distant recurrence, higher than the rate found in our cohort, though the number of patients undergoing primary intracranial treatment with placement of carmustine wafers in our cohort was significantly smaller.

A retrospective review by Ene et al. in 2016 evaluated 14 patients receiving implantation of carmustine wafers during surgical resection of brain metastasis [23]. All patients had received a prior form of treatment with subsequent recurrence prompting craniotomy and carmustine wafer placement. 50% of patients had tumor recurrence with mean time to progression of 2.5 years, and 5 patients died prior to publication with a mean overall survival of 2.9 years. This cohort found that patients > 53 years old had a longer progression free survival than those < 53 years old, and no variable affected overall survival. Though their PFS and OS reported are higher than our current cohort, this could be secondary to evaluating a significantly smaller retrospective cohort, or shorter median follow up time (18.7 months).

Radiosurgery is a commonly used adjuvant modality for brain metastasis treatment following maximum safe total resection to combat microscopic tumor cells outside of the resection cavity. Unfortunately, the brain has limited tolerance to radiation therapy, and over treatment with radiation can lead to complications such as tumor necrosis. More modern studies have documented the incidence of radiation necrosis following re-irradiation of brain metastases ranging from 13.4% to 38.4% [24, 25]. Within these studies, the largest risk factors were determined to be tumor size, radiation dose, and presence of prior treatment with WBRT. A retrospective review of 31 patients undergoing placement of carmustine wafers for treatment of brain metastasis following radiosurgical failure was performed by Mu et al. in 2015 to evaluate alternatives to re-irradiation [21]. Median overall survival for these patients was 10 months, with 45% dying of CNS-tumor related cause. Local control for these patients at 1 year was 70%, with 16% undergoing further salvage therapy, as compared to our cohort with a 1 year survival of approximately 60% and 8% of patients who received carmustine wafers following SRS failure undergoing further neurosurgical salvage therapy. Additionally, median overall survival in our cohort was comparatively prolonged at 23 months, which may be secondary to the homogeneity of patients undergoing primary or secondary treatment for brain metastasis in our cohort. When accounting only for patients who had previously undergone radiotherapy, 6 (16%) had local recurrence in our cohort, similar to the rates described in the Mu et al. cohort, and 5 of 38 previously treated with SRS had local recurrence in 1 year, giving a 1-year local progression rate of 13%. Comparatively, local progression rates for patients undergoing reirradiation following radiosurgical failure are reported up to 39%, with OS ranging from 7.9 to 24 months in some series [26].

### Limitations

While we describe one of the largest prospectively evaluated cohorts of patients undergoing implantation of GLIADEL wafers during resection of brain metastasis with the goal of describing progression free and overall survival, our population size is limited. Additionally, we have no prospective control group to compare, and thus can only provide descriptive evaluation of our results. Further large prospective or randomized controlled trials are needed to draw significant conclusions regarding benefit on overall and progression free survival. Next, data from the VIGILANT prospective registry was made available for analysis after completion in 2023. Therefore, emerging additions to the arsenal of targeted therapy used to treat intracranial metastases were not included in the current study.

## Conclusion

Patients described in this series represent a particularly challenging population—those with brain metastases that had either recurred after stereotactic radiosurgery or were initially too large for resection followed by radiosurgery to the resection cavity to have a high probability of success. For these individuals, effective salvage or alternative local therapies are extremely limited. By focusing on this heterogenous high-risk group, our results support the observed safety, feasibility, and local control within this cohort for whom few other options exist, regardless of prior treatment.

Placement of GLIADEL wafers for treatment of brain metastases may be associated with encouraging rates of overall survival and of local progression free survival compared to historical rates described in the literature, particularly in those who have failed radiosurgery as initial management. GLIADEL was well tolerated, with few adverse events attributable to GLIADEL placement.

## Supplementary Information

Below is the link to the electronic supplementary material.

Supplementary Material 1

## Data Availability

https://clinicaltrials.gov/study/NCT02684838.
