# Supplementary material for: Carmustine-releasing wafers for brain metastasis: single institution experience and results of the VIGILANT registry
Source: J Neurooncol. 2026 Nov 11;180(2):46. doi: 10.1007/s11060-026-05871-2 (PMC13755031; doi:10.1007/s11060-026-05871-2)
Supplement: Supplementary file 1 [file 11060_2026_5871_MOESM1_ESM.docx]

Supplementary Table 1. Adverse events by system organ class

| **System organ class** | **Patients, n (%)*** | **Events, n (%)*** | **Fatal events, n** |
| --- | --- | --- | --- |
| **Any adverse event** | **43 (91.5)** | **136** | **25** |
| **Neoplasms (primary, metastatic, systemic)** | **31 (66.0)** | **35 (25.7)** | **17** |
| CNS disease progression (growth, recurrence, leptomeningeal disease) | 20 (42.6) | 24 (17.6) | 7 |
| Systemic neoplasm progression/metastasis | 8 (17.0) | 8 (5.9) | 7 |
| Primary solid tumor progression | 1 (2.1) | 1 (0.7) | 1 |
| New intracranial metastases | 2 (4.3) | 2 (1.5) | 1 |
| **Nervous system disorders** | **31 (66.0)** | **33 (24.3)** | **0** |
| Seizure | 5 (10.6) | 6 (4.4) | 0 |
| Encephalopathy | 4 (8.5) | 4 (2.9) | 0 |
| Intracranial vasogenic edema | 4 (8.5) | 4 (2.9) | 0 |
| Cerebrospinal fluid leakage | 3 (6.4) | 3 (2.2) | 0 |
| Hemiplegia/limb weakness, new or worsening | 4 (8.5) | 5 (3.7) | 0 |
| Hydrocephalus | 1 (2.1) | 1 (0.7) | 0 |
| Aphasia | 1 (2.1) | 1 (0.7) | 0 |
| Cerebral cyst | 1 (2.1) | 1 (0.7) | 0 |
| Cerebral infarction | 1 (2.1) | 1 (0.7) | 0 |
| Dizziness | 1 (2.1) | 1 (0.7) | 0 |
| Otherwise unspecified neurologic deficit/disorder | 6 (12.8) | 6 (4.4) | 0 |
| **Infectious Disease** | **15 (31.9)** | **19 (14.0)** | **1** |
| Pneumonia | 8 (17.0) | 10 (7.4) | 0 |
| Sepsis | 3 (6.4) | 5 (3.7) | 1 |
| Urinary tract infection | 2 (4.3) | 2 (1.5) | 0 |
| Empyema | 1 (2.1) | 1 (0.7) | 0 |
| Wound infection | 1 (2.1) ** | 1 (0.7) ** | 0 |
| **Respiratory disorders** | **12 (25.5)** | **12 (8.8)** | **1** |
| Respiratory failure | 5 (10.6) | 5 (3.7) | 1 |
| Pulmonary embolism | 2 (4.3) | 2 (1.5) | 0 |
| Aspiration | 2 (4.3) | 2 (1.5) | 0 |
| Bronchopleural fistula | 1 (2.1) | 1 (0.7) | 0 |
| Hypoxia | 2 (4.3) | 2 (1.5) | 0 |
| **Gastrointestinal disorders** | **7 (14.9)** | **7 (5.1)** | **1** |
| Colitis | 1 (2.1) | 1 (0.7) | 0 |
| Dysphagia | 2 (4.3) | 2 (1.5) | 0 |
| Gastrointestinal bleeding | 1 (2.1) | 1 (0.7) | 0 |
| Nausea | 1 (2.1) | 1 (0.7) | 0 |
| Vomiting | 1 (2.1) | 1 (0.7) | 0 |
| Esophageal Fistula | 1 (2.1) | 1 (0.7) | 1 |
| **Cardiac disorders** | **4 (8.5)** | **5 (3.7)** | **0** |
| Atrial fibrillation (with and without RVR) | 2 (4.3) | 3 (2.2) | 0 |
| Coronary artery disease | 1 (2.1) | 1 (0.7) | 0 |
| Tachyarrhythmia | 1 (2.1) | 1 (0.7) | 0 |
| **Psychiatric disorders** | **3 (6.4)** | **5 (3.7)** | **0** |
| Mental status changes | 2 (4.3) | 4 (2.9) | 0 |
| Confusion/Delirium | 1 (2.1) | 1 (0.7) | 0 |
| **Injury and other procedural complications** | **7 (14.9)** | **10 (7.4)** | **5** |
| Radiation necrosis | 2 (4.3) | 2 (1.5) | 0 |
| Pneumocephalus | 1 (2.1) | 1 (0.7) | 0 |
| Car accident | 1 (2.1) | 1 (0.7) | 0 |
| Impaired wound healing | 1 (2.1) ** | 1 (0.7) ** | 0 |
| Sudden or unexplained death | 5 (10.6) | 5 (3.7) | 5 |
| **Metabolism and nutrition disorders** | **4 (8.5)** | **4 (2.9)** | **0** |
| Hyponatremia | 2 (4.3) | 2 (1.5) | 0 |
| Hyperglycemic hyperosmolar nonketotic syndrome | 1 (2.1) | 1 (0.7) | 0 |
| Hypernatremia | 1 (2.1) | 1 (0.7) | 0 |
| **Other system conditions (Renal, Vascular, etc.)** | **6 (12.8)** | **6 (4.4)** | **1** |
| Acute kidney injury | 2 (4.3) | 2 (1.5) | 0 |
| Left-sided hearing loss | 1 (2.1) | 1 (0.7) | 0 |
| Hyperbilirubinemia | 1 (2.1) | 1 (0.7) | 0 |
| Stevens-Johnson syndrome | 1 (2.1) | 1 (0.7) | 1 |
| Deep vein thrombosis | 1 (2.1) | 1 (0.7) | 0 |

*Patient percentages were calculated out of a total of 47 patients enrolled in the study. Event percentages were calculated out of the total number (136) adverse events.

**Indicates events that were denoted as possibly related to implantation of Gliadel
